# High-Sensitivity and Low-Hysteresis Porous MIM-Type Capacitive Humidity Sensor Using Functional Polymer Mixed with TiO_2_ Microparticles

**DOI:** 10.3390/s17020284

**Published:** 2017-02-02

**Authors:** Ming-Qing Liu, Cong Wang, Nam-Young Kim

**Affiliations:** Radio Frequency Integrated Circuit Center, Kwangwoon University, Nowon-gu, Seoul 139-701, Korea; tzjzlmq@kw.ac.kr

**Keywords:** porous MIM-type capacitive humidity sensor, functional polymer, TiO_2_ microparticles

## Abstract

In this study, a high-sensitivity and low-hysteresis porous metal–insulator–metal-type capacitive humidity sensor is investigated using a functional polymer mixed with TiO_2_ microparticles. The humidity sensor consists of an optimally designed porous top electrode, a functional polymer humidity sensitive layer, a bottom electrode, and a glass substrate. The porous top electrode is designed to increase the contact area between the sensing layer and water vapor, leading to high sensitivity and quick response time. The functional polymer mixed with TiO_2_ microparticles shows excellent hysteresis under a wide humidity-sensing range with good long-term stability. The results show that as the relative humidity ranges from 10% RH to 90% RH, the proposed humidity sensor achieves a high sensitivity of 0.85 pF/% RH and a fast response time of less than 35 s. Furthermore, the sensor shows an ultra-low hysteresis of 0.95% RH at 60% RH, a good temperature dependence, and a stable capacitance value with a maximum of 0.17% RH drift during 120 h of continuous test.

## 1. Introduction

Humidity sensors have been widely used in our daily lives and have become increasingly important in different applications such as meteorology, agriculture, smart homes, medical equipment, and biotechnology [1,2,3,4]. Various types of humidity sensors are available, based on different measurement principles such as resistive, capacitive, optical, acoustic, and thermal techniques [5,6,7,8]. Among these several types of humidity sensors, the capacitive devices are preferred owing to their high sensitivity, linearity, accuracy, fast response time, and negligible temperature [9,10,11,12]. Most of these capacitive sensors are based on the dielectric changes in the sensing layer upon water vapor uptake as a measure of the water vapor content.

In capacitive humidity sensors, the material of the humidity-sensing layer is one of the most important components. Many types of materials, such as electrolytes [13,14], ceramics [15,16,17], and polymers [18,19,20,21], have been proposed for humidity sensors by exploiting the variations in their electrical parameters. In particular, polymers have been extensively investigated for use as a sensing material in capacitive humidity sensors owing to their good hygroscopic and dielectric properties. Organic polymers consist of a unit structure of repeated macromolecules, and most of them are carbon-hydride compounds or their derivatives. Polymers contain micro-pores for water vapor condensation, and some of the measurable physical properties change because of water absorption. Moreover, it is very easy to coat and pattern polymers owing to their photo-sensibility. Polymeric humidity sensors have been widely studied in research and applied in industry for over 40 years. Organic polymer-based humidity sensors suffer from large hysteresis between absorption and desorption of moisture as well as low response. Hysteresis is a major problem for capacitive humidity sensors; it may lead to deformation of the polymer and thus affect the sensor performance adversely.

The current paper proposes a high-sensitivity and low-hysteresis metal–insulator–metal (MIM) capacitive humidity sensor using a well-designed porous top electrode and a functional polymer mixed with TiO_2_ microparticles. The porous top electrode increases the contact area between the functional polymer sensing layer and the environment, resulting in high sensitivity and fast response time. A functional polymer is proposed by mixing an organic polymer with TiO_2_ ceramic-based microparticles to achieve ultra-low hysteresis and enhance the humidity-sensing range. The humidity characteristics of the sensor have been tested in various relative humidity conditions. The use of a well-designed porous top electrode and a functional polymer mixed with TiO_2_ microparticles can improve the properties of the humidity sensor; thus, high sensitivity, low hysteresis, fast response time, and wide humidity-sensing range can be realized.

## 2. Structure and Fabrication

**Figure 1:**
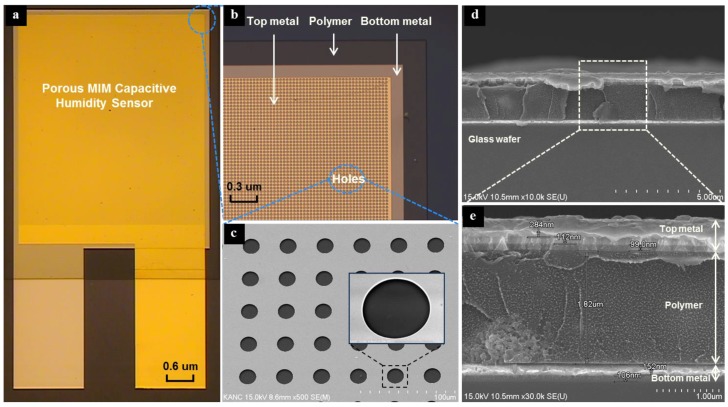
(**a**) Micrographs of the implemented porous MIM-type capacitive humidity sensor, (**b**) an enlarged view of the capacitive humidity sensor, (**c**) SEM images of the enlarged holes, (**d**) a cross-sectional view of the MIM-type capacitive humidity sensor, and (**e**) an enlarged cross-sectional view.

The porous MIM-type capacitive humidity sensor, having a size of 4 mm × 5 mm, consists of a glass substrate, a bottom electrode, a functional polymer film as a sensing layer, and a top electrode with holes. Figure 1a,b show micrographs of the implemented capacitive humidity sensors. The holes in the top electrode are provided to increase the surface area and hence enhance the diffusion of water vapor into the functional polymer sensing layer. Different hole diameters and spaces between adjacent holes are designed to realize high sensitivity and short response time. A novel functional polymer mixed with TiO_2_ microparticles is proposed for the humidity-sensing layer; it exhibits low hysteresis and good linearity to humidity. The proposed functional polymer is composed of pyromellitic dianhydride (PMDA)–oxydianiline (ODA), which is formed from the polyamic acid precursor of PMDA and ODA with mixed TiO_2_ microparticles to form a composite material in a weight ratio of 1:1.5, which can fully mix together and good for the water vapor absorption and desorption to realize a high sensitivity and low hysteresis. The average grain size of the TiO_2_ microparticles is less than 0.1 µm; they are of the rutile type with a dielectric constant of 3.9. Titanium dioxide is a very useful additive, which can improve the hysteresis behavior and humidity-sensing range of the polymer for humidity detection.

**Figure 2:**
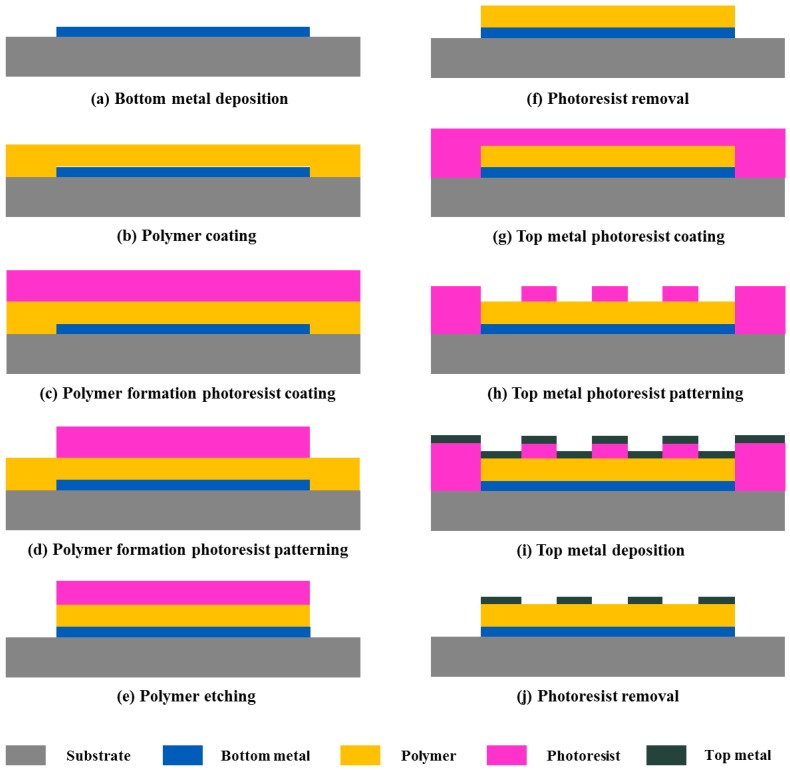
Fabrication process of porous MIM-type capacitive humidity sensor: (**a**) bottom metal deposition, (**b**) polymer coating, (**c**) polymer formation photoresist coating, (**d**) polymer formation photoresist patterning, (**e**) polymer etching, (**f**) photoresist removal, (**g**) top metal photoresist coating, (**h**) top metal photoresist patterning, (**i**) top metal deposition, and (**j**) photoresist removal.

The substrate used in the fabrication is a 6 in. glass wafer. The fabrication process of the porous MIM-type capacitive humidity sensor is shown in Figure 2 as follows: (a) An e-beam-evaporated Ti/Pt layer is deposited at 1000/2000 Å to obtain the bottom electrode. (b) A 1.8-µm-thick polymer is spin-coated on the surface of the bottom electrode. Then, hard baking is performed at 100 °C for 10 min and at 210 °C for 30 min to remove 95% of the water in the polymer, thereby promoting its sensitivity. (c) A negative photoresist, specifically, a 4.8-µm-thick DNR-L300-40 (Dongjin Semichem Co., Ltd., Seoul, South Korea), is spun onto the surface of the polymer layer at 3000 rpm for 40 s. (d) Following exposure by a chrome mask, the patterns are transferred to the photoresist, which is developed by an AZ300MIF developer (AZ Electronic Materials USA Corp., NJ, USA) and cleaned via O_2_/N_2_H_2_ plasma descum step. (e) The polymer is etched using CF_4_/O_2_ in a BMR inductively coupled plasma (ICP) asher with a chamber temperature of 81 °C and ICP power of 900 W for 3 min. Some TiO_2_ residue remains after this etching process. (f) A TiO_2_ residue removal method was previously proposed by our team [22]. We found that ultrasonic-treated acetone liquid can be used to completely remove the residues and photoresist simultaneously. (g) A positive photoresist, which is a 2-µm-thick GXR601 (AZ Electronic Materials, Korea, Seoul, Korea), is spun onto the surface of the polymer layer at 4500 rpm for 40 s. (h) The photoresist is then patterned to create holes; it is developed in a 2.38% TMSH developer and cleaned via O_2_/N_2_H_2_ plasma descum step. (i) A 1000/1000/3000 Å-thick Ti/Pt/Au metal layer is formed by e-beam evaporation, which is used as the top electrode. (j) Finally, a lift-off process is used to create the top electrode on the patterned photoresist layer.

The fabricated porous MIM-type capacitive humidity sensors are shown in Figure 1. The cross-sectional views of the humidity sensor pattern and the enlarged holes on the top electrode are investigated using a scanning electron microscope (SEM) (S-4300SE, Hitachi Co., Ltd., Tokyo, Japan), as shown in Figure 1c–e.

## 3. Results and Discussion

**Figure 3:**
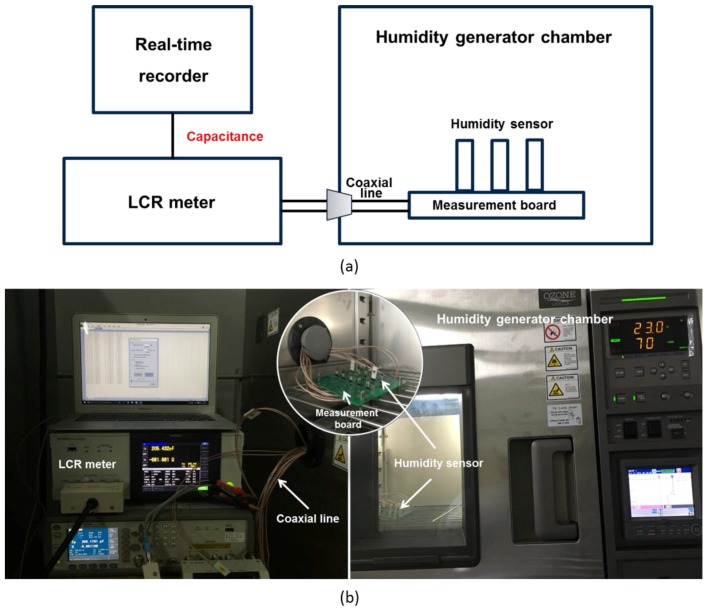
Humidity sensor measurement setup: (**a**) diagram and (**b**) photograph. The humidity sensors are fixed on the measurement board and placed into the humidity generating chamber. Further, they are connected to the LCR meter using coaxial lines to minimize the noise and obtain the measured capacitances.

Figure 3 shows a diagram and photograph of the measurement setup for the capacitive humidity sensor. The capacitances of the sensors are measured using an LCR meter (IM3536, HIOKI E. E. Corp., Ueda, Japan) in a humidity-generating chamber (PDL-3J, ESPEC Corp., Osaka, Japan) at 1 kHz, 1 V_pp_; the relative humidity (RH) is used to fit experimental results. The chamber fluctuations in terms of temperature and humidity are ±0.3 °C and ±2.5% RH, respectively. First, we fix the sensors on the measurement board and place it into the humidity-generating chamber. Then, we use coaxial lines, which have low online losses, to connect the measurement board to the LCR meter through a hole on the side wall of the chamber using a good sealant. The varying capacitances can be recorded in real time on the computer through a USB connection with the LCR meter. Before the measurement process, the fabricated humidity sensors were dried and stored in a desiccator to maintain the initial absorbed moisture level close to zero. The chamber humidity was increased from 10% RH to 90% RH and then decreased back to 10% RH in steps of 10% RH; the measurements were taken at 30 min intervals to ensure full sorption/desorption of water vapor.

### 3.1. Sensitivity

Capacitive humidity sensors respond to humidity variations by varying their dielectric permittivity, and this variation is directly proportional to the ambient vapor changes. The ideal capacitance *C* of the MIM capacitive humidity sensor is expressed as

(1) $$C=\varepsilon_{0}\varepsilon_{r}\frac{A}{d}$$

where $\varepsilon_{0}$ is the permittivity of vacuum, $\varepsilon_{r}$ is the relative permittivity of the functional polymer mixed with TiO_2_ microparticles, *A* is the effective sensing area of the humidity sensor, and *d* is the thickness of the sensing layer. The permittivity of the polymer changes proportionally with the high dipole moments of water molecules. Therefore, this humidity change is directly detected by measuring the changes in the capacitance. The relative permittivity of the polymers in room conditions is approximately 5, while that for pure water is far greater (around 80). When water vapor is absorbed by polymers, the apparent permittivity value will increase, resulting in a linear increase in capacitance with relative humidity.

**Figure 4:**
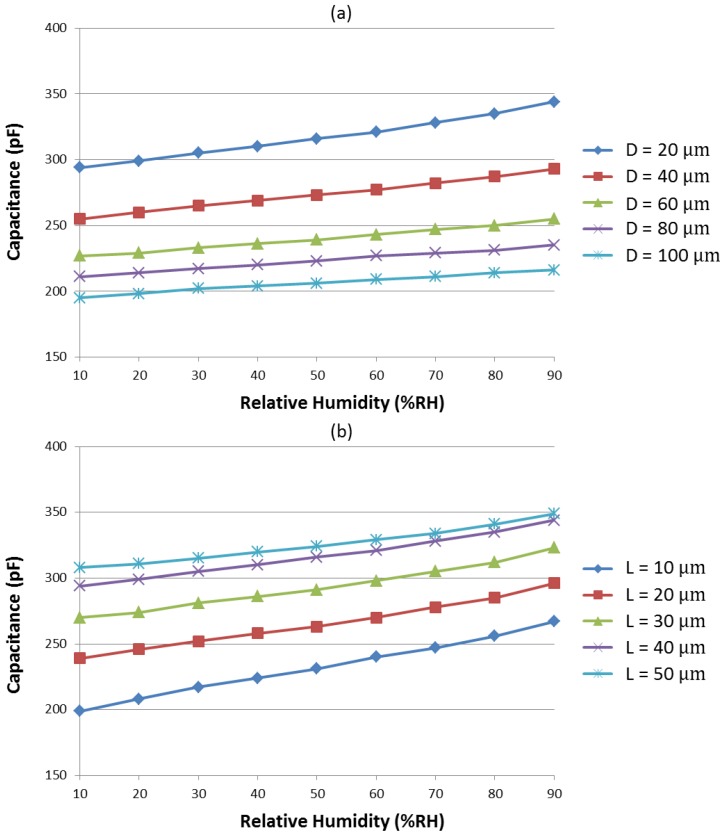
Measured capacitance of the humidity sensors during relative humidity changes from 10% RH to 90% RH with (**a**) different hole diameters and (**b**) different space between the adjacent holes.

Figure 4 shows the capacitance of the humidity sensors with different hole diameters (*D*) and spaces between the adjacent holes (*L*). The sensors show linear outputs with high sensitivity using the functional polymer mixed with TiO_2_ microparticles. The sensitivity *S* of the humidity sensors can be expressed as

(2) $$S=\frac{C_{90}-C_{10}}{90-10}\;(pF/\%\;RH)$$

where *C*_90_ and *C*_10_ denote the capacitance obtained at 90% RH and 10% RH, respectively. The 90 and 10 values are the highest and lowest RH values in the variation range, respectively. The sensitivity of the humidity sensor with different hole diameters (20 µm to 100 µm) is shown in Figure 4a; all other conditions remain the same, and the space between holes is fixed at 40 µm. The sensor with a smaller hole diameter can achieve high sensitivity because more holes exist on the top electrode with the same area. Therefore, the contact area between the polymer and water vapor is enlarged, and high sensitivity is obtained. The sensitivity of the humidity sensors with different spaces between adjacent holes (10 µm to 50 µm) is shown in Figure 4b; all other conditions remain the same, with the hole diameter fixed at 20 µm. For the same reason, the sensor with smaller space between adjacent holes can achieve high sensitivity. In summary, a small hole diameter and a smaller space between adjacent holes imply wider distribution of the hole density at the top of the electrode and greater surface area access for the water vapor. This condition allows more water molecules to be randomly adsorbed to form a multi-layer structure of condensed water, thereby improving the sensitivity of the humidity sensors. Finally, we choose a sensor with a hole diameter of 20 µm and a space between adjacent holes of 10 µm, which can realize high sensitivity of 0.85 pF/% RH. The ratio between the top metal and the holes is around 1:0.62.

### 3.2. Hysteresis

Hysteresis is a critical drawback of capacitive humidity sensors. It may cause deformation of the polymer owing to water clusters and consequently influence the sensor performance. To reduce the hysteresis, a functional polymer is proposed by mixing an organic polymer with TiO_2_ ceramic-based microparticles. The role of the TiO_2_ microparticles can be explained using an ionic conduction model [23]. The water molecules are available for adsorption on the surfaces of the TiO_2_ microparticles to form hydroxyl groups. When more water is further adsorbed, each water molecule is hydrogen-bonded to two hydroxyls; then, a liquid-like layer of hydrogen-bonded water molecules is formed, in which each water molecule is only singly bonded to a hydroxyl group. This process could effectively suppress the water clusters in the polymer, resulting in low hysteresis.

**Figure 5:**
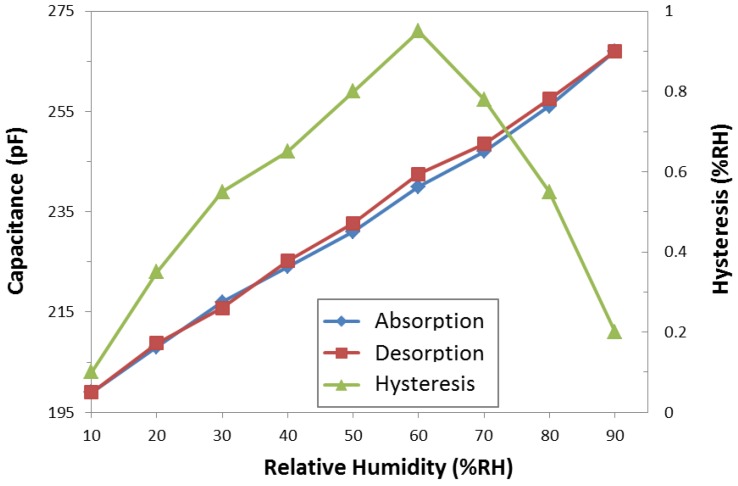
Hysteresis characteristics of the humidity sensors in relative humidity variation from 10% RH to 90% RH.

The capacitance variation with the humidity is measured to investigate the hysteresis behavior of the humidity sensor, as shown in Figure 5. The hysteresis of the highest-sensitivity humidity sensor with a hole diameter of 20 µm and space between adjacent holes of 10 µm is evaluated at 23 °C. The sensors are placed in a humidity chamber, and the humidity is increased from 10% RH to 90% RH and then decreased to 10% RH in steps of 10% RH. The humidity sensor exhibits maximum hysteresis *H* of 0.95% RH at 60% RH, which is calculated by

(3) $$H=\frac{C_{D60}-C_{A60}}{S}\;(\%\;RH)$$

where *C*_D60_ and *C*_A60_ are the capacitance values measured at 60% RH in the desorption and absorption process, respectively. Figure 5 shows that the hysteresis increases at the beginning, reaches its maximum value at 60% RH, and then decreases with a further increase in the RH. The maximum hysteresis of 0.95% RH is far below one of the best commercial standard values (Vaisala; 2% RH ± 0.3% RH). This result is a good indication that functional polymer mixed with TiO_2_ microparticles reduces the hysteresis value.

### 3.3. Response Time

**Figure 6:**
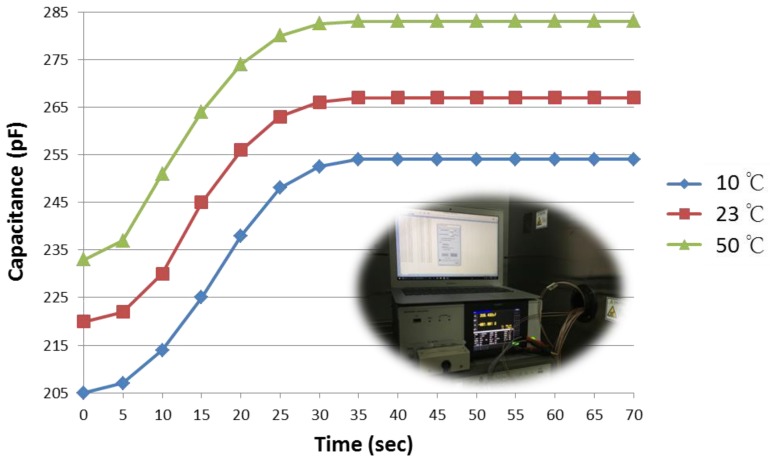
Response time measurement result of the humidity sensors from room environment to 90% RH at 10 °C, 23 °C, and 50 °C.

The response time is verified by the capacitance variation of the humidity sensor when the ambient humidity is abruptly changed from room conditions to 90% RH. The experimental setup was constructed as follows. The chamber was kept at 90% RH steadily. The humidity sensors were put into the 90% RH chamber at room humidity level through the hole in the chamber. The LCR meter was connected to the humidity sensor throughout the entire process, and it measured the response time at the 90% RH point of the final steady-state capacitance value after an abrupt capacitance change. The response times of the same humidity sensor at three different temperatures (10 °C, 23 °C, and 50 °C) were measured. Figure 6 shows that the response times are less than 35 s owing to the enlarged contact area between the polymer and water vapor through the intensive hole structures at the top electrode, which can achieve good adsorption of water vapor and fast subsequent diffusion into the humidity-sensing film. Temperature has no effect on the response time of the humidity sensor.

### 3.4. Temperature Dependence

**Figure 7:**
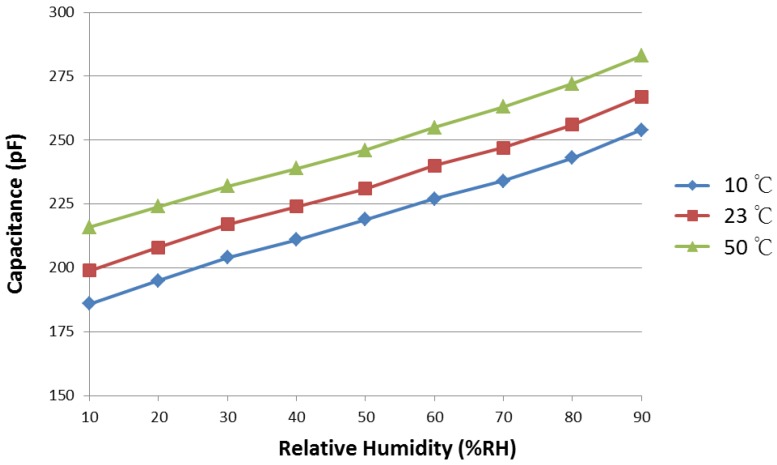
Temperature dependence of the humidity sensor measured at 10 °C, 23 °C, and 50 °C.

To determine whether the temperature influences the sensitivity of the humidity sensor, the capacitance changes at three different temperatures (10 °C, 23 °C, and 50 °C) are measured. Figure 7 shows the corresponding humidity-capacitive characteristics of the sensors. The capacitance of the humidity sensors across the entire humidity range generally increases with an increase in the ambient temperature. The magnitude of the capacitance indicates the dielectric property and the polarization property of the sensing layer. The thermal movement of molecules is an internal cause for the material polarization. As the temperature increases, the thermal movement of the molecules becomes strong, and the increase in polarization results in an increase in the capacitance value [24]. However, the slope of the curves remains virtually unchanged at different temperatures, which means that the temperature does not influence the sensitivity of the humidity sensor. This result is a good indication for temperature-independent sensor applications.

### 3.5. Stability

**Figure 8:**
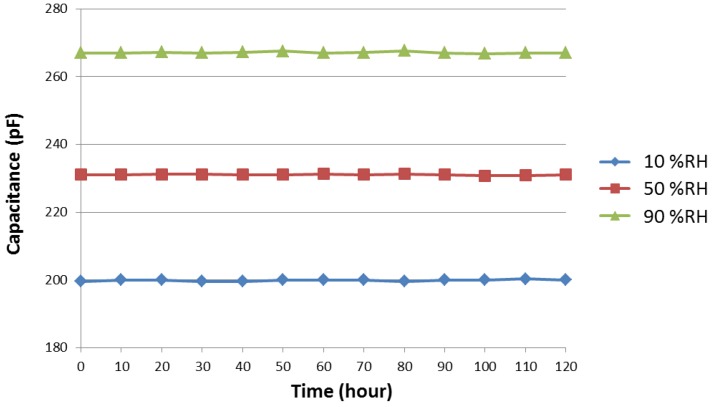
Stability property of the humidity sensor at 10% RH, 50% RH, and 90% RH during 120 h of continuous testing.

The long-term stability is tested to investigate the practical value of the humidity sensor. The sensors are placed in an ambient chamber for 120 h at 10% RH, 50% RH, and 90% RH. Figure 8 shows that the sensors maintain stable capacitance values, verifying the good long-term stability of the functional polymer mixed with TiO_2_ microparticles. The magnitude of the drift of sensor capacitance is converted into the apparent changes in relative humidity, *D*, which is calculated by [25]

(4) $$D=\frac{C\left(\mathrm{meas}\right)-C(\mathrm{init})}{S}\;(\%\;RH)$$

where C(meas) is the measured capacitance after the sensor was exposed to 10% RH, 50% RH, and 90% RH atmosphere at a certain time, and C(init) is the initial capacitance before the test. The maximum drift value obtained under different relative humidity values is 0.17% RH. Therefore, from a practical perspective, the sensor has achieved significant stability and is a very promising candidate for a commercially available humidity sensor.

Table 1 compares the humidity sensor using functional polymer with TiO_2_, organic polymer without TiO_2_, and the other referenced sensors. The proposed humidity sensor, which employs a functional polymer mixed with TiO_2_ microparticles, realizes high sensitivity, low hysteresis, fast response time, and wide humidity-sensing range.

## 4. Conclusions

A novel humidity-sensitive layer composed of a functional polymer mixed with TiO_2_ microparticles and a porous top electrode structure was designed to improve the performance of MIM-type capacitive humidity sensors. This work demonstrated a high sensitivity of 0.85 pF/% RH and a fast response time of less than 35 s, which can be achieved by the hole structure on the top electrode. Furthermore, this research achieved an ultra-low hysteresis of 0.95% RH and a wide humidity-sensing range from 10% RH to 90% RH, which can be realized by the functional polymer mixed with TiO_2_ microparticles. Finally, a good temperature-independent and stable capacitance value with a maximum of 0.17% RH drift was shown to be sufficient for use in a reliable humidity sensor for various applications.

## Figures and Tables

**Table 1:** Comparison between the proposed humidity sensor and the referenced sensors.

Ref.	Polymer mixed with Ceramic	Sensitivity (pF/% RH)	Hysteresis (% RH)	Response Time (s)	Sensing Range (%)
[18]	✗	0.2	3	-	10–90
[19]	✗	0.38	2	60	30–90
[20]	✗	0.38	2.63	216	10–90
[21]	✗	0.35	1.3	40	30–90
Our polymer without TiO_2_	✗	0.36	1.7	45	10–90
**Our polymer with TiO_2_**	**✓**	**0.85**	**0.95**	**35**	**10–90**
